# Electronic and electrochemical viral detection for point-of-care use: A systematic review

**DOI:** 10.1371/journal.pone.0258002

**Published:** 2021-09-30

**Authors:** Solen Monteil, Alexander J. Casson, Samuel T. Jones

**Affiliations:** 1 Department of Materials, School of Natural Sciences, University of Manchester, Manchester, United Kingdom; 2 The Henry Royce Institute, Manchester, United Kingdom; 3 Department of Electrical and Electronic Engineering, School of Engineering, University of Manchester, Manchester, United Kingdom; University of Waterloo, CANADA

## Abstract

Detecting viruses, which have significant impact on health and the economy, is essential for controlling and combating viral infections. In recent years there has been a focus towards simpler and faster detection methods, specifically through the use of electronic-based detection at the point-of-care. Point-of-care sensors play a particularly important role in the detection of viruses. Tests can be performed in the field or in resource limited regions in a simple manner and short time frame, allowing for rapid treatment. Electronic based detection allows for speed and quantitative detection not otherwise possible at the point-of-care. Such approaches are largely based upon voltammetry, electrochemical impedance spectroscopy, field effect transistors, and similar electrical techniques. Here, we systematically review electronic and electrochemical point-of-care sensors for the detection of human viral pathogens. Using the reported limits of detection and assay times we compare approaches both by detection method and by the target analyte of interest. Compared to recent scoping and narrative reviews, this systematic review which follows established best practice for evidence synthesis adds substantial new evidence on 1) performance and 2) limitations, needed for sensor uptake in the clinical arena. 104 relevant studies were identified by conducting a search of current literature using 7 databases, only including original research articles detecting human viruses and reporting a limit of detection. Detection units were converted to nanomolars where possible in order to compare performance across devices. This approach allows us to identify field effect transistors as having the fastest median response time, and as being the most sensitive, some achieving single-molecule detection. In general, we found that antigens are the quickest targets to detect. We also observe however, that reports are highly variable in their chosen metrics of interest. We suggest that this lack of systematisation across studies may be a major bottleneck in sensor development and translation. Where appropriate, we use the findings of the systematic review to give recommendations for best reporting practice.

## 1 Introduction

Viruses are a significant global health problem. The difficulty associated with detecting viruses results in increased transmission, as well as delayed and potentially more expensive treatment. The ongoing SARS-CoV-2 pandemic has highlighted the urgent global need for faster viral detection methods. Even before the pandemic, there was much research interest in faster and more accurate virus detection, and particularly detection that can be carried out at the Point-of-Care (PoC) [1].

The current standard for virus identification is via cell culture, referred to as virus isolation [2, 3]. Virus isolation is reliable, however it can take anywhere from days to weeks, and requires trained personnel as well as expensive equipment. The gold standard remains Polymerase Chain Reaction (PCR) [4, 5]. By amplifying target nucleic acid in the presence of fluorescent dyes, such as SYBR green, semi-quantitative results can be obtained in a few hours. However, PCR requires precise thermal cycling and therefore generally expensive equipment. Other sensing targets are also available, such as detecting virons (individual virus molecules), or detecting viral antigens or antibodies produced by the person against the virus. For example Enzyme-Linked Immunosorbent Assay (ELISA) is a plate-based method to detect molecules (such as antigens or antibodies) that is routinely used for the detection of Human Immunodeficiency Virus (HIV) and Dengue Virus (DENV) infections [6, 7]. Nevertheless, plate-based conventional ELISA tests are typically lengthy and require technical skills to perform.

PoC sensors are used to detect the analyte of interest, while overcoming the issues of traditional detection methods such as cost, time, equipment, and the need for trained personnel. PoC sensors are designed to be used in locations such as a physician’s office, at home, or in the field. Such sensors aim to give rapid results that would allow the user to receive immediate treatment, care, or begin other interventions, such as self-isolation. For example, lateral flow tests have been available for many decades and have been widely used in out-of-clinic settings for self-testing for SARS-CoV-2 [8]. However, they can confirm only the presence or absence of the target analyte, they do not provide any quantification, and as they are based on visual inspection can be misread by lay-users.

To overcome these limitations, there has been much interest in electronic PoC sensors which can provide benefits in assay time, limit of detection, sensitivity, and quantification. Many sensing approaches have been explored such as optical (*e.g*. colorimetry, fluorimetry, surface plasmon resonance); mechanical (*e.g*. quartz crystal microbalance); electronic (*e.g*. Field Effect Transistor (FET), impedance); and electrochemical (*e.g*. voltammetry, amperometry, electrochemical impedance spectroscopy), aiming to measure a number of different analytes (*e.g*. DNA, antigens, antibodies). Benefitting from advances in the semiconductor industry and mass-consumer electronics, electronic and electrochemical detection approaches are currently an extremely active area of research. Here we focus on electronic and electrochemical detection systems as one of the most promising approaches to achieve cheap, rapid and quantitative results, that can be readily scaled down to make the sensor truly portable. (Optical and mechanical PoC sensors have been previously reviewed in [9–11] and will not be considered here.).

Here we present a systematic review into the state of the art in electronic and electrochemical viral sensors. Given the importance of virus detection, and the high need for rapid PoC systems, a number of review papers on virus detection have been presented recently. For example, Khan *et al*. [1] gave a critical review covering electrochemical impedance biosensors, electrochemical immunosensors, and DNA based biosensors. de Eguilaz *et al*. [12] gave a narrative review focusing on electrochemical detection for PoC devices. Similar recent reviews, mainly focusing on SARS-CoV-2, have been given in a number of papers [12–21], as well as recent reports on devices such as those from Seo *et al*. and Chaibun *et al*. [22, 23]. To our knowledge however, all of the reviews to date have been critical, scoping or narrative reviews [24]; that is, reviews with the selection of papers primarily guided by the authors. This reflects the technical origins of the papers, in fields where such reviews are very common. However it falls short of systematic evidence collection required in the clinical domain. In the clinical domain the gold standard is a systematic review following the 2020 PRISMA guidelines [25]. This sets rigorous requirements for study inclusion, exclusion and reporting. Given the clinical importance of PoC biosensors for virus detection, and to facilitate connections between the different stakeholder communities, we believe it is imperative to provide a systematic review following formal procedures and standards for evidence synthesis, which is not addressed by current reviews. We focus on comparing the main differences in detection method (voltammetry, impedance and field effect transistors) and target (antibody, antigen, nucleic acid and virion), using assay times and reported Limits of Detection (LoD) as the evaluation criteria. We believe that this gives a different overview of the state of the art, and the relative prominences and performance of different approaches, compared to previously presented reviews.

## 2 Results

**Fig 1:**
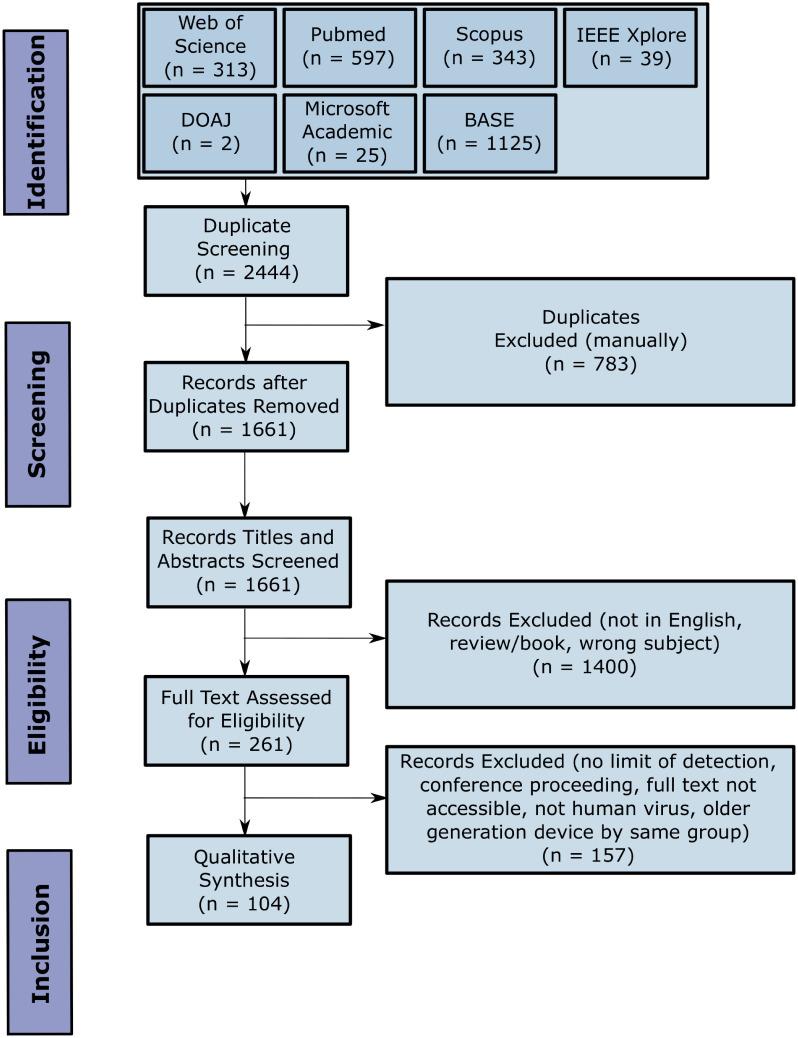
PRISMA flowchart. PRISMA flowchart detailing the systematic review process.

In order to conduct a systematic review, we searched 7 databases with an identical search query, initially yielding 1661 papers (Fig 1). After removing repeats, all but original research articles detecting human viruses, and including only those reporting a limit of detection, we identified 104 papers reporting an electronic or electrochemical sensor for viral detection at the Point-of-Care (PoC). To compare the Limit of Detection (LoD) across papers it was necessary to convert detection limits to a common unit. Nanomolar (nM) is used here, as over 80% of the papers analysed in this review were either already in nM or could be converted to nM (see Methods 5.3), and this unit relates to the number of targets detected rather than their mass. The conversion between copies/mL and nM, requiring multiplication by a constant, is independent of the molecular weight making the two equivalent to one another. However, it should be noted that the composition of the sensor input samples differs widely, with some studies using complex clinical samples such as blood and saliva, while others use homogeneous solutions of purified target analyte in buffer. The assay times used in this review are either reported or inferred based on the methods section of reported papers (*vide infra*). To compare LoDs and assay times, we grouped the papers by both detection method, and by target analyte, as both strongly influence the performance of the sensor. For our review, we assume the reader has basic familiarity with different detection approaches (*e.g*. cyclic voltammetry *vs*. differential pulse voltammetry) and virus sensing terminology but provide a brief introduction where appropriate.

### 2.1 Detection methods

**Fig 2:**
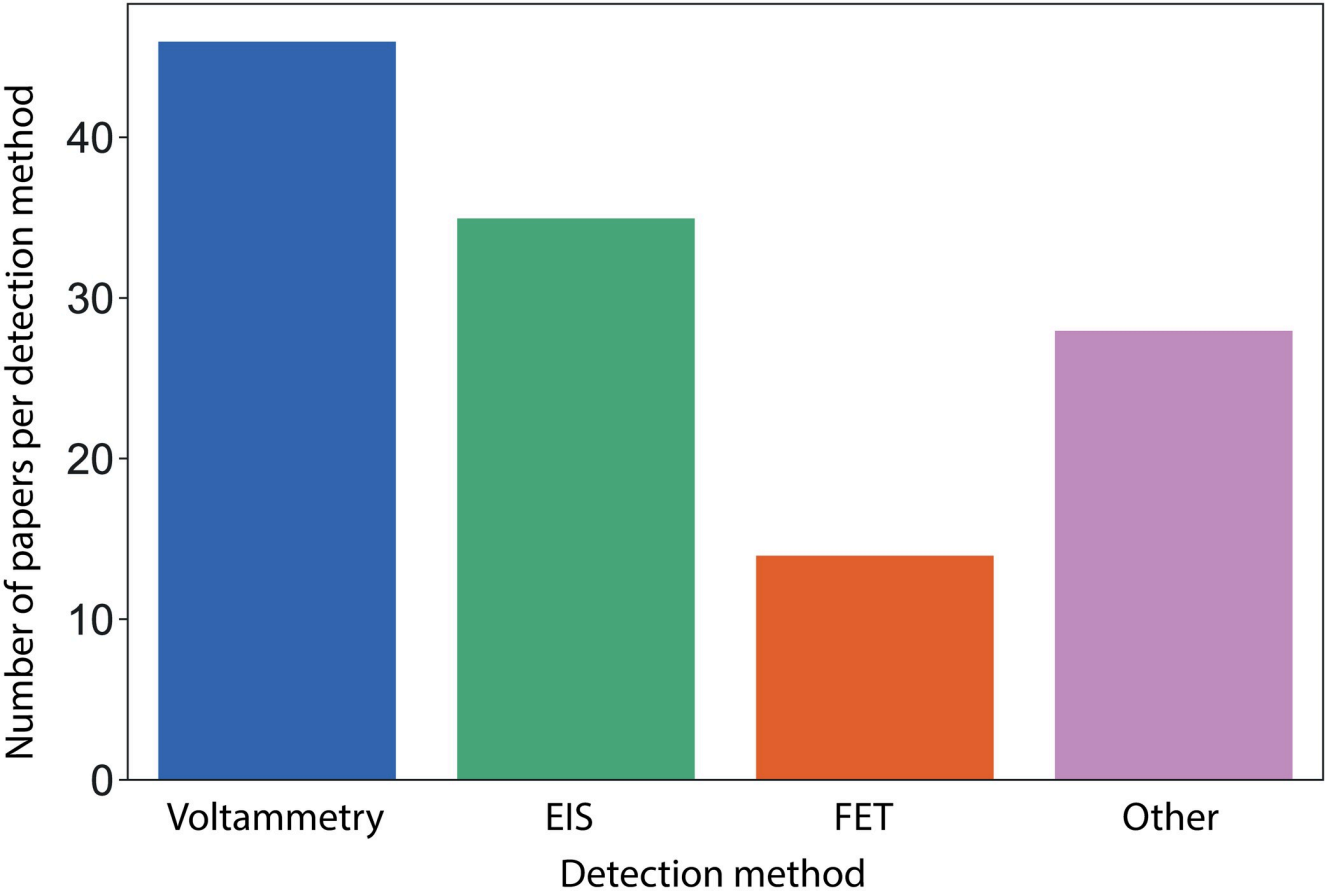
Number of papers selected for this review divided by detection method. Voltammetry (blue), EIS (green), FET (orange), other (purple). EIS: Electrochemical Impedance Spectroscopy, FET: Field Effect Transistor.

Within the 104 papers retained for review, 18 reported more than one method to detect the viral pathogen (*e.g*. the use of voltammetry and impedance within the same device). As shown in Fig 2, voltammetry (including Cyclic Voltammetry (CV), Differential Pulse Voltammetry (DPV), and Square Wave Voltammetry (SWV)) was the primary detection method in 46 papers (54% use CV, 28% use DPV, 22% use SWV). This is followed by Electrochemical Impedance Spectroscopy (EIS, 35 papers) and then Field Effect Transistors (FETs, 14 papers). Other methods have also been used such as amperometry, nanopores, and ion exchange membranes, although in lesser proportions. These have been captured within the *other* category in Fig 2.

Based upon these frequencies of use, the following sections describe each electrical detection method in turn and the reported LoDs and assay times. The results are summarised in Table 1.

**Table 1:** Summary table of response time and LoD per detection method.

Detection method	Response time (min)				Limit of Detection (nM)				Ref.
	Min	Max	Median	Std. dev.	Min	Max	Median	Std. dev.	
CV	2.0	210.0	32.0	61.9	2.8 × 10^−12^	3.2 × 10^1^	1.1 × 10^−3^	8.0 × 10^0^	[26–50]
DPV	15.0	720.0	32.0	199.0	2.9 × 10^−12^	1.8 × 10^1^	5.1 × 10^−2^	5.7 × 10^0^	[32, 42, 47, 51–60]
SWV	16.0	93.0	40.0	24.0	3.3 × 10^−8^	2.6 × 10^2^	1.0 × 10^−3^	8.2 × 10^1^	[61–70]
EIS	2.0	360.0	30.0	63.3	2.8 × 10^−12^	2.7 × 10^1^	1.3 × 10^−5^	5.8 × 10^0^	[26–29, 34–37, 39, 41, 42, 45, 49, 50, 65, 71–90]
FET	0.0*	120.0	7.5	36.0	1.2 × 10^−12^	1.0 × 10^0^	1.0 × 10^−6^	3.0 × 10^−1^	[91–104]

Std. dev.: standard deviation, CV: Cyclic Voltammetry, DPV: Differential Pulse Voltammetry, SWV: Square Wave Voltammetry, EIS: Electrochemical Impedance Spectroscopy, FET: Field Effect Transistor. (* indicates 10 milliseconds.)

#### 2.1.1 Voltammetry

Broadly, voltammetry operates by applying an electrical potential to an electrode (called the working electrode) where the target analyte binds. The resulting current is then measured, with this changing when different levels of analyte are present. Within this, many different forms of voltammetry are possible, varying depending on how the electrical potential is changed over time. Voltammetry is particularly suited to PoC biosensor applications as printed electrodes can be used, easily integrated into printed electronics platforms with scalable manufacturing.

Within our considered papers Cyclic Voltammetry (CV) is the most reported method, with Differential Pulse Voltammetry (DPV) and Square Wave Voltammetry (SWV) also widely used. CV allows the characterisation of redox processes such as the presence of intermediate steps during oxidation-reduction reactions, or the reversibility of a reaction, while DPV and SWV are of particular interest for the detection of pathogens due to their low sensitivity to capacitive current resulting in increased sensitivity at low target concentrations [105, 106].

From our study, the lowest LoDs using voltammetry for nucleic acid detection are: 2 × 10^−10^ nM (120 copies/mL) (CV), 3×10^−8^ nM (2 × 10^4^ copies/mL) (SWV), 3 × 10^−12^ nM (1.72 copies/mL) (DPV). The fastest device across all targets was shown by Singhal *et al*., who reported an incubation time of just 35 seconds (with EIS to confirm hybridisation; time quoted in Table 1 also takes into account measurement time, see Methods section) [34]. Their system detected nucleic acids from Chikungunya virus with a LoD of 3.4 nM (2 × 10^12^ copies/mL). It should be noted however, that the assay time has high variability between different voltammetric sensors due to different purification and hybridisation times, not only the measurement times. For example, in 2019 Moço *et al*. developed a RNA sensor for Zika virus (ZIKV) using DPV as the sensing method [52]. The team immobilised ZIKV nucleic acid probes on graphite electrodes modified with electrochemically reduced graphene oxide and polytyramine-conducting polymer. The response was evaluated by DPV in the presence of a ferrocyanide redox couple. The sensor was able to detect ZIKV genomic RNA at concentrations in the zeptomolar range (10^−12^ nM, 1.72 copies/mL) in 20 minutes.

Overall the reported performance of voltammetry based sensors is highly variable. There is a wide range of detection limits (from zeptomolar to nanomolar), and response times (from 2 minutes to over 3 hours).

#### 2.1.2 Electrochemical impedance spectroscopy

Unlike voltammetry, Electrochemical Impedance Spectroscopy (EIS) is not based on changing the voltage, but on changing the frequency of the applied voltage. The impedance of the electrode and bound target is measured by applying a small excitation signal (AC potential, typically ∼1–10 mV) and measuring the current that flows. The response to a small sinusoidal potential will be a sinusoidal current of the same frequency, with a magnitude and phase depending on the impedance of the sample being analysed (assuming the overall system is linear). These factors are measured and plotted. Impedance is a useful tool for pathogen detection due to its sensitivity, label-free sensing and low power, although software fitting of the impedance curve is required which is not always accurate [107, 108]. Some papers make use of similar but different approaches, for example measuring only changes in capacitance or resistance rather than measuring the complex impedance at different frequencies.

As with the voltammetry based sensors, LoDs and response times vary widely across the range of EIS sensors in this study. As shown in Table 1, response times vary from two minutes up to six hours (with a standard deviation of 63 minutes) and LoD from zM to tens of nM. As an illustrative example, in 2019 Baek *et al*. developed a sensor for human norovirus detection using EIS (with CV as a cross-validation measurement). The device could detect virions in concentrations as low as 3 × 10^−12^ nM (1.7 copies/mL) in under 35 minutes [36].

#### 2.1.3 Field Effect Transistors (FETs)

FETs are comprised of a *source* and a *drain* electrode, linked by a semi-conductor *channel*, with a *gate* above the channel to control current flow through it. Either the gate (used in 3 out of 14 papers considered here) or the channel (used in 7 out of 14 papers considered here) can be used as a binding site to change the current flow depending on the amount of analyte present. Transconductance is the ratio between the drain current and the gate-source voltage (minus the transistor’s threshold voltage) and changes in transconductance are the commonly reported detection mechanism.

An important characteristic of FET based sensors is their sensitivity, with a median LoD in Table 1 of the order of 10^−6^ nM. This compares to 10^−5^ nM for EIS, 10^−3^ nM for CV and SWV, and 10^−2^ nM for DPV. From our included papers the FET sensor with the lowest LoD detects SARS-CoV-2 spike protein (antigen) in concentrations as low as 1 × 10^−12^ nM [103]. The reported FET sensors are also rapid. In Table 1 the median response time was under 8 minutes for FETs, compared to 30, 32, 32, 40 minutes for EIS, DPV, CV and SWV respectively. The fastest device is a SARS-CoV-2 sensor developed by Xian *et al*., detecting the spike protein in 10 ms down to 450 fM [101].

However, these figures mask the fact that in the papers identified in this review, the FETs are primarily used with antigens (often commercial). The reported measures therefore do not necessarily take into account the time required for pre-treatment of clinical samples. Recently, FETs have been tested with complex biological samples such as saliva or serum [103], which will significantly improve the scope of such sensors for real world use. Also, not all FET sensor designs are compatible with existing microelectronics industry routes to scale-up manufacturing. Many academic FET detection systems are manufactured by trained personnel in a cleanroom environment, which is expensive and can impact sensor to sensor reproducibility. For example, many state of the art FET sensors use channels made out of graphene or carbon nanotubes, which can have high device to device variation [109, 110]. Afsahi *et al*. showed that this issue can be overcome by using a commercial production technique, packaging chemical vapor deposition grown graphene into ceramic carriers (compatible with pre-existing electronics production foundries) [94, 111]. FETs are the most recent category of detection approach, and have clear potential benefits compared to voltammetry and impedance based approaches, providing that the scalability issues can be addressed.

### 2.2 Targets

**Fig 3:**
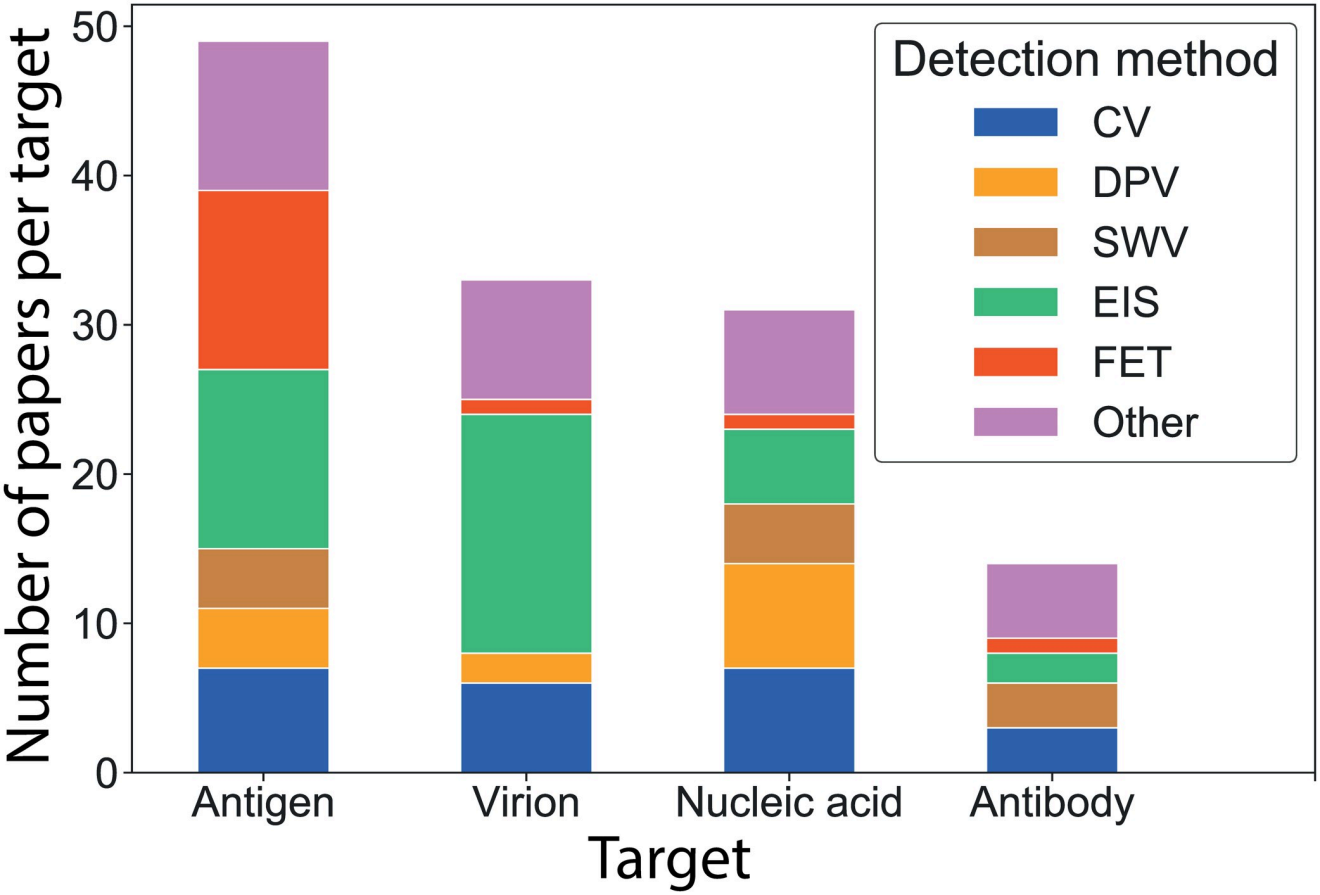
Detection method used per target. Number of papers selected for this review divided by target (antigen, virion, nucleic acid and antibody), and coloured by detection method: Voltammetry (blue), EIS (green), FET (orange), other (purple).

The previous section investigated the impact of electronic and electrochemical detection methods on reported sensor performances. Also critically affecting performance is the target analyte to be detected. Within the 104 papers retained for review, PoC sensors are based principally on the same targets as conventional laboratory testing, namely virions, nucleic acids, antibodies, or antigens. We found that most PoC sensors focus on the detection of antigens (39%), followed closely by virions (26%), nucleic acids (26%), and in lesser proportions antibodies (12%), with a few papers detecting 2 different targets, Fig 3.

Interestingly, Fig 3 shows that CV is used across all types of targets, whereas SWV is not used with virions and DPV is more prevalent for nucleic acids (50% of DPV papers use nucleic acids). Similarly 12 out of 14 FET based sensors were detecting antigens. In the current state of the art, the FET advantages (discussed above) in LoD and response time come at the cost of a reduced range of target analytes.

**Fig 4:**
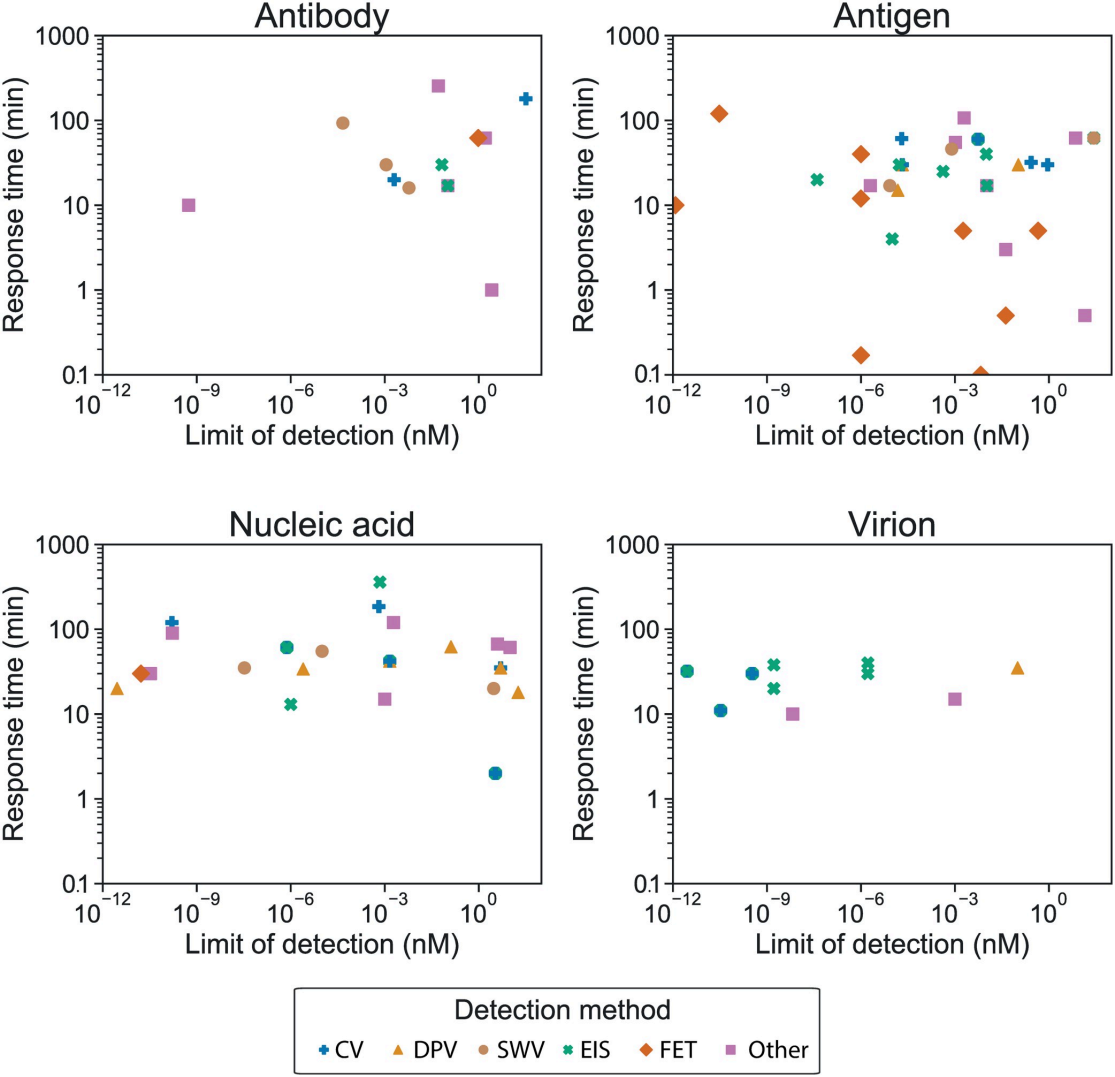
Sensor properties represented as response time vs. detection limit, by detection method. Only papers from our review presenting a limit of detection in nM or convertible to nM are included in this graph. The results for different targets are shown in different sub-plots: antibody (A), antigen (B), nucleic acid (C) and virion (D); separated by detection method: CV (blue plus), DPV (gold triangle), SWV (brown circle), EIS (green cross), FET (orange diamond), other (purple square).

A summary of the response time and LoDs per target is given in Table 2, with the two-way trade-off plotted in Fig 4. This shows the response time in minutes and the limit of detection in nanomolars by target, including only sensors reporting a LoD in nM or convertible to nM. Comparisons between the LoD for each analyte category should not be made, as the clinical significance is very different for the same amount of different analytes.

**Table 2:** Summary table of response time and LoD per target.

Target	Response time (min)				Limit of Detection (nM)				Ref.
	Min	Max	Median	Std. dev.	Min	Max	Median	Std. dev.	
Antibody	1.0	255.0	30.0	75.6	5.5 × 10^−10^	3.2 × 10^1^	6.7 × 10^−2^	8.8 × 10^0^	[30, 33, 44, 61, 62, 68, 83, 89, 102, 112–115]
Antigen	0.0*	120.0	25.0	28.8	1.2 × 10^−12^	2.6 × 10^2^	1.9 × 10^−3^	4.2 × 10^2^	[31, 32, 35, 38, 41, 48–50, 55, 57, 65, 68–72, 83–85, 88–99, 101, 103, 104, 116–122]
Virion	0.5	720.0	30.0	128.9	2.8 × 10^−12^	1.0 × 10^−1^	1.7 × 10^−9^	2.8 × 10^−2^	[26, 27, 36, 37, 39, 45, 51, 56, 73–75, 77–82, 87, 90, 101, 123–129]
Nucleic acid	2.0	360.0	42.0	75.0	2.9 × 10^−12^	1.8 × 10^1^	1.0 × 10^−3^	4.0 × 10^0^	[29, 34, 40, 42, 43, 46, 47, 52–54, 59, 60, 63, 64, 66, 67, 76, 86, 100, 130–137]

Std. dev.: standard deviation. (* indicates 10 milliseconds.)

Fig 4 clearly shows that all targets demonstrate a very wide range of LoDs, spanning 9 orders of magnitude in all cases. Similarly, response times span at least two orders of magnitude for all targets other than virions. (Enzyme targets are excluded here as only one paper, [28], reported the detection of enzymes.) The performance of current PoC sensors for each type of analyte is discussed in turn below.

#### 2.2.1 Antibodies

When the body has been exposed to pathogens it develops antibodies in response. Antibodies can be divided into 5 main classes of immunoglobulins (Ig)—IgA, IgD, IgE, IgG and IgM; each class having different mechanisms of action and targets [138]. For example, IgM are the first antibodies to be made during an infection, whereas IgG take time to form after an infection and have a better specificity to the pathogen [139, 140]. Detecting antibodies can determine if a person is currently infected or has been infected in the past [83, 141].

In our study, antibody based PoC sensors have a limit of detection ranging from 6 × 10^−10^ nM to 32 nM, and take between a minute and four hours (see Table 2). Wang *et al*. developed the sensor with the lowest limit of detection (6 × 10^−10^ nM) using capacitance to detect Zika virus antibodies in under 10 minutes [113]. The fastest antibody test was made by Morin *et al*. to detect HIV. The sensor was a nanopore, needing only 1 minute to detect antibodies in concentrations of 3 nM [112].

Targeting antibodies gives the benefit of being able to infer information on the time of infection [83, 141]. However, for some viruses such as hepatitis C (HCV), antibodies take several weeks (8–11) to be produced to a detectable level [142]. Therefore antibodies may not be the best targets to detect all viral infections quickly, compared to using viron or other targets, which do not require time for the body to produce a response. The vast majority of reviewed papers report only the time from the sample being available as the detection time, masking the practical time that is actually required overall.

#### 2.2.2 Antigens

Viral antigens are proteins encoded by the viral genome present on the virus surface, and can be recognised by the host immune system. Antigens often provide the virion with host-cell binding ability.

In the current study, antigen sensors have a limit of detection ranging from 10^−12^ nM (via FET detection) to 260 nM (via SWV detection), and take from a few seconds to two hours. The device with the lowest LoD is a SARS-CoV-2 spike protein sensor developed by Guo *et al*., reaching single molecule detection in 10 minutes [103]. Another device reached single molecule detection with a different architecture, a HIV sensor developed by Macchia *et al*. for the p24 capsid protein, detecting concentrations as low as 2±1 proteins per mL, equivalent to 3 × 10^−11^ nM [98]. The fastest antigen sensor is another FET based one, detecting the SARS-CoV-2 spike protein in milliseconds from spiked saliva (Xian *et al*.) [101].

However, an issue with antigen detection is potential cross-reactivity. Cross-reactivity is when an antibody designed to detect a given antigen also has high affinity towards another antigen. For example, Zika virus (ZIKV) is a member of the flavivirus family (e.g. Dengue virus, Japanese encephalitis virus), and the antigen similarity between ZIKV, Japanese encephalitis virus and Dengue virus makes their specific detection challenging. ELISA kits for ZIKV detection therefore have high false positive rates (sometimes as high as 59% [143]), as previous infection by, or vaccination against, a flavivirus can result in a positive test. To overcome this issue, highly specific antibodies need to be developed with explicit *challenge* tests performed to check for cross-reactivity (see [94] for an example).

To target antigens the sensor site is activated with molecules for the antigen to bind to. From our review, these probes are usually antibodies (76%) and sometimes aptamers (10%) or peptides (6%). However, the way the antibody binds to the sensor surface has a great impact on target detection, due to orientation issues sometimes leading to a loss of biological activity [144]. Antibodies also make expensive probes, due to their challenging production [145].

Further, the detection of only the antigen requires isolation and purification of the viral surface proteins first, which is not ideal in a PoC setting. Thus, while the results in Table 2 show that antigen sensors have a fast response time, this may be discarding the sample preparation time required. The reporting standards between different studies are highly variable making it difficult to estimate this preparation information in all cases.

#### 2.2.3 Virions

Virions are individual virus molecules, designed to transmit their genome into host cells. Virions are made of nucleic acid surrounded by a protein coat (capsid) and sometimes a lipid membrane (envelope). The virion surface is coated with proteins allowing binding to host cells. As a result, PoC sensors based on the detection of virions require limited pre-treatment such as the need for extraction/amplification.

In the current study, virion sensors have a limit of detection ranging from 3 × 10^−12^ nM (1.7 copies/mL) (via CV and EIS detection) to 10^−1^ nM (6 × 10^10^ copies/mL) (via DPV detection), taking between 30 seconds and 12 hours. The sensor with the lowest LoD detects noroviruses using peptides as probes on a gold electrode, taking over 30 minutes (LoD of 3 × 10^−12^ nM, 1.7 copies/mL) [36]. The fastest device is based on capacitance measurements to detect influenza A, giving a result in 30 seconds with a LoD of 0.25 pg/mL [124].

The main advantage of aiming to detect virus particles directly is the absence of a need for pre-treatment; developing a device insensitive to the additional proteins present in bodily-fluid samples provides a substantial usability advantage to reduce assay time and complexity. However, to detect viruses the probe is commonly an antibody, having the disadvantages highlighted above such as cost and loss of activity upon attachment. 65% of papers in our study detecting virions used antibody probes, although other attachment methods exist such as aptamers.

#### 2.2.4 Nucleic acids

Viral particles contain nucleic acids, either DNA or RNA, and viruses of the same genus such as ZIKV and DENV are distinguishable from differences in their genome sequences. Using nucleic acid targets thus gives a highly specific technique to identify viruses. Nucleic acid approaches have been shown to allow effective discrimination between viruses of the same genus with a single-base mismatch specificity [146].

General nucleic acid detection requires amplification to increase the amount of nucleic acid material present. Since the 1990s, nucleic acid amplification has become commonplace, making the detection of nucleic acids easier. Recently isothermal amplifications have been developed that are better suited to PoC sensors than traditional PCR as they work at a single temperature. Removing thermal cycling can help achieve a smaller size and a reduced power consumption for use in a PoC device.

In the current study, nucleic acid sensors have a limit of detection ranging from 3 × 10^−12^ nM (1.72 copies/mL) (via DPV detection) to 18 nM (10^13^ copies/mL) (DPV), and take between 2 minutes and 6 hours. For example Moço *et al*. achieved a LoD of 3 × 10^−12^ nM (1.72 copies/mL) for the detection of ZIKV RNA in 20 minutes, using DPV on real samples from infected patients [52]. The fastest device was developed by Singhal *et al*., detecting Chikungunya virus DNA in a few minutes with an optimised hybridisation time of 35 seconds, and a LoD of 3.4 nM (2 × 10^12^ copies/mL) [34].

Nucleic acids are found inside the virion, protected by the capsid and sometimes the envelope. Thus to detect nucleic acids, the protective layers need to be removed, often chemically or thermally. However, most chemical agents needed to access the nucleic acids have to be washed before the detection step as they can impact the results [147]. Another issue with nucleic acids is with double stranded genomes, which need to be denatured to bind to the probe. Using heat to release and denature nucleic acids is a good way to limit the number of steps, however it may not be compatible with low power miniature PoC devices, especially battery powered devices.

## 3 Discussion and implications for future virus sensor design

Examining the many PoC sensors identified in this article, grouping them either by detection method or by target provides useful insights into sensor performance. From this analysis it is clear that many other parameters influence the limit of detection and assay time. For example two papers from Venkatesh *et al*. and Zhao *et al*. use very similar approaches but report widely different performances [30, 33]. They both describe the detection of the same antibody (IgG against HCV) in serum by indirect ELISA, and use cyclic voltammetry. In both papers an alkaline phosphatase (ALP)-labelled secondary antibody binds to the antibody of interest, and p-aminophenyl phosphate (PAPP) is catalysed to p-aminophenol (PAP) with ALP. PAP is then oxidised, generating electrons that create a signal. However, the device from Venkatesh has a LoD of 32 nM whereas the one from Zhao has a LoD of 0.002 nM. The response times are 180 seconds and 20 minutes, respectively.

Certainly, differences can be noted between the two papers. Different electrodes are used (screen printed gold for Venkatesh and screen printed carbon for Zhao), and a different molecule to bind the antigen to the electrode surface (gold binding peptide (GBP) and 3-amino-propyldimethylethoxysilane (APDES) with glutaraldehyde (GA), respectively). Such differences in assay times and LoDs suggest that targets and detection methods are not enough to determine the figures of merit of a sensor.

Whilst some trends are apparent from the analysis of performance, either by detection method or target, there are a number of additional factors which may influence results. For instance, there are numerous experimental set-ups, such as the tethering of the probe to the electrode surface/material, and the shape and enhancements of the probe, and various electrode materials, rendering meaningful comparison very challenging.

This is compounded by the variability in reporting found across different studies. Our review shows that the reporting of electronic and electrochemical viral sensors lacks homogeneity, with a general need to focus more on actual clinical needs. This is evidenced very directly from our inclusion criteria. A simple literature research for virus detection returns thousands of results, but the relatively modest inclusion criteria of requiring a LoD to be reported renders the vast majority of papers to be excluded.

Focusing on the LoD, usually the LoD is accepted as either 3.3× *s*/*m* from the calibration curve (with *m* being the slope of the calibration curve, and *s* the standard deviation of the lowest concentration); 3 times the standard deviation of the blank (sample containing no target) response; or the smallest signal that gives signal-to-noise ratio of 3:1 or 2:1 (in different studies) [148, 149]. However, even within these varying definitions LoD lack of homogeneity in their reporting. Sometimes the LoD is defined as 3× *s*/*m*, which although is a minor difference in coefficient, can result in variable LoDs. Some calibration curves are only made of 3 or 4 points, making the fit much less reliable. Sometimes the LoD is simply defined as the lowest concentration detected by the sensor. Based upon the best practice seen in our reviewed papers we recommend for the future reporting of devices that authors base their calibration curves on at least 5 different concentrations, in at least 3 replicates (ideally 6) from different devices, and suggest authors explicitly state by which method they obtained the LoD.

Going beyond this, to express the limit of detection of a developed sensor, authors use several different units, making the direct comparison of LoDs from different sensors challenging. In the 104 papers analysed in this review, the different units used were nM, ng/mL, copies/mL, HAU/mL (hemagglutinin units per mL), PFU/mL (plaque forming units per mL), TCID50/mL (50% tissue culture infective dose per mL). Units assessing the infectivity (PFU/mL, TCID50/mL) cannot be linked to units that measure the concentration of targets (nM, ng/mL, copies/mL, HAU/mL). All of these are divorced from the clinical requirements: identifying what different diagnosis or treatment options are enabled at different levels of performance and whether an improved LoD actually gives any practical benefit for real collected clinical samples. In order to allow better comparison, the units in this review have been converted to nM where possible, with the conversion equations given in Methods.

In our view nM is more favourable than mg/mL as it directly relates to the number of targets detected and does not depend on molecular weight. This allows for determination of which method (target or device) gives signal for the fewest targets. This should not be confused with biological concentrations present during, or required for, infection. Comparing biologically relevant LoDs would require separating all targets, as well as all viruses, and would not allow for comparisons across devices to be made. In addition, there are a lack of standards for some targets, for example HIV antibodies do not yet have an international standard, leading to lack of reliability when comparing different assays. The aim of this review is not to provide a biologically-relevant quality assessment of individual sensors, instead it is to compare across all devices and draw conclusions on what makes the most sensitive and fastest sensors.

Similar comments apply when reporting the detection speed, with variable practice in the reporting of any pre-processing steps required. A general standard for each virus and target, as well as a better communication between relevant parties and common units would be of tremendous help in building trust in sensor performance. Reproducibility of sensors from device-to-device and user-to-user also needs to be reported more if devices are to move beyond the proof-of-principle stage, with metrics such as the coefficient of variation (the ratio of standard deviation to the mean, also known as Relative Standard Deviation (RSD)) reported. There is scope for a terminology to reflect the current status of a new sensor development; whether it is a proof of principle; a feasibility study using a larger range of testing to demonstrate the utility of moving forwards with an approach; a pilot with real users to inform the design of a larger scale trial; or a larger scale in-the-wild test. Comparing different sensor designs efficiently would allow the field to progress more rapidly, which is urgently needed.

Another key parameter to consider is selectivity, which is paramount for real-world use of sensors, beyond just sensitivity. However, this is difficult to compare across a wide range of devices and viruses targeted. This is due in part to challenges associated with selection of viruses used to determine selectivity, as well as rapidly changing viral serotypes, for example. Although not compared herein, the selectivity of individual sensors captured by this review are available in S1 Table.

Based upon our experience in collating and harmonising this review we recommend future papers to report at least the following results:

- Limit of detection
- Linear range
- Sample-to-result time
- Selectivity to relevant viruses
- Sensor lifetime and storage conditions
- Device-to-device variability
- Whether the sensor is single-use, and if not report recovery time and variability.

Where possible these should be with clinical samples. While we understand that not every research group may have access to clinical samples and that they are not necessarily needed to develop new sensors, their use is a very important validation step. Further, the above are in addition the electronic parameters, around physical size and power consumption for PoC use, cost, and device usability and evidence of co-design with actual users.

## 4 Conclusion

Here we have compared 104 papers describing point-of-care electronic and electrochemical viral sensors, by limits of detection as well as by reported or inferred assay times. Due to the large array of units used to report limits of detection it was necessary to convert all values to a single unit (nanomolar), where possible. Voltammetry was the most used detection method (44%), followed by impedance spectroscopy (33%) and field effect transistors (13%). The latter showed the fastest assay time (median under 8 minutes), although with most manuscripts using commercial antigen samples. Field effect transistors also have the lowest limit of detection with a median of 1 pM, with several of them reaching single molecule detection. In terms of target analytes, we find that the fastest to detect are antigens, having a median assay time of 25 minutes.

Through our systematic review we find that field effect transistors show the most promise for future devices, dependant upon scaled-up manufacturing becoming available. Recently, more rigorous testing of field effect transistors with non-commercial antigens or the use of complex biological samples has been shown, which will significantly improve the scope of such sensors for real world use. However, there is still a lot of work to be done before the adoption of these devices in a clinical setting becomes widespread.

Although analysing the assay times and limits of detection provides some guidance towards the sensor design, more parameters need to be taken into account, such as the electrode material and complexity of real world samples. Moreover, considerations such as cost, shelf-life, and power consumption are important when considering PoC sensors for real world use. The benefit of performing a formal systematic review is that it demonstrates the need for a better coherence between papers reporting on viral sensors, with regards to units used and measurement times and reproducibility. This is urgently needed to help the scientific community better understand what makes the best sensors to build improved devices. Intensive research and consideration are still required to push the technology further through using large numbers of patient derived samples to test the reliability and reproducibility of PoC sensors.

## 5 Methods

### 5.1 Literature search methods

This systematic review was conducted as per the guidelines outlined by 2020 Preferred Reporting Items for Systematic Reviews and Meta-analysis (PRISMA) statement [25]. The PRISMA 2020 checklist is included as S1 Appendix. The review was not pre-registered in PROSPERO.

#### 5.1.1 Search strategy

Relevant studies were identified by conducting a search of current literature using 7 databases: PUBMED, Web of Science, Scopus, Microsoft Academic, IEEE Xplore, Directory of Open Access Journals (DOAJ), and Bielfeld Academic Search Engine (BASE). The search strategy used for a comprehensive search was as follows: (virus OR viral) AND (sensor OR sensing OR detection OR biosensor) AND (electronic OR electrochemical OR impedance OR FET OR voltammetry) AND (POC OR “point of care” OR “total analysis system” OR TAS OR portable OR bedside). An initial search was conducted on 05/05/2020, second search on 28/10/2020 excluding Microsoft Academic due to significant changes in their search algorithm, and a third search extending the search query to “field effect transistors” on 01/06/2021.

#### 5.1.2 Inclusion and exclusion criteria

- Virus: Studies had to report the detection of at least one human virus strain. Only viruses capable of human to human transmission were selected for this review, excluding plant and animal viruses, as well as avian influenza.
- Study design: Only primary research studies were included. Reviews, case-reports, books, conference proceedings, testing of commercial devices, and editorial reports were excluded. Where the same group had reported different generations of the same device, only the most recent paper was selected.
- Outcomes: Studies had to report a limit of detection in order to be included in this review.

#### 5.1.3 Selection process

All studies identified using the specified search strategy from the 7 databases were first screened using the title and the abstract. Abstracts were included or excluded based on the criteria defined above (apart from the absence of LoD which was excluded only after whole paper screening). Only publications written in English were included. Abstracts which met the criteria were then re-screened using the full-text article. These were assessed for reporting of outcomes prior to final inclusion in the review.

#### 5.1.4 Data extraction

Relevant information was extracted and details of each study were then summarised in a table. A copy of the full results is included as S1 Table. The following data was extracted: virus, detection method, electrode, probe, target, labelling, medium, limit of detection, range, selectivity, response time, recovery time, lifetime, and validity (principally RSD). Where the total assay time was not reported but methods were detailed, assay times were inferred by adding incubation time with 1 minute per wash and 1 minute for detection.

### 5.2 Study selection

Fig 1 summarises the number of studies screened at each stage of the selection process. A total of 1661 studies were identified from searching the databases. From these, 1400 were excluded and 261 included based on the title and abstract. A further 157 articles were excluded based on the full text for the reasons detailed before. Finally, the remaining 104 studies were included in this review and analysed.

### 5.3 Calculations

1. From copies/mL to nM:
  $$\begin{array}{c} \text{concentration}\;\text{in}\;\text{nM}=\frac{\text{number}\;\text{of}\;\text{copies}\;\text{per}\;\text{mL}}{\text{Avogadro}’\text{s}\;\text{number}}\times \frac{\text{nM}}{\text{M}}\times \frac{\text{mL}}{\text{L}} \end{array}$$ (1)
  which is equivalent to
  $$\begin{array}{c} \text{concentration}\;\text{in}\;\text{nM}=\frac{\text{number}\;\text{of}\;\text{copies}\;\text{per}\;\text{mL}}{6.022}\times 10^{-11} \end{array}$$ (2)
2. From mg/mL to nM: The conversion depends on the molecular weight of the target.
  Examples: M_w_(IgG) = 150 kDa, M_w_(NS1) = 55 kDa.
  1 kDa ≈ 10^3^ g/mol
  $$\begin{array}{c} \text{concentration}\;\text{in}\;\text{nM}=\frac{\text{concentration}\;\text{in}\;\text{mg/mL}}{{\text{M}}_{\text{w}}\;\text{in}\;\text{kDa}}\times \frac{\text{nM}}{\text{mM}} \end{array}$$ (3)
  which is equivalent to
  $$\begin{array}{c} \text{concentration}\;\text{in}\;\text{nM}=\frac{\text{concentration}\;\text{in}\;\text{mg/mL}}{{\text{M}}_{\text{w}}\;\text{in}\;\text{kDa}}\times 10^{6} \end{array}$$ (4)

## Supporting information

S1 Appendix PRISMA 2020 checklist. (PDF)

S1 Table Data extracted from all papers selected for this review. (XLSX)

S1 Graphical abstract (TIFF)
