# Supplementary figures and images for: Electronic and electrochemical viral detection for point-of-care use: A systematic review
Source: PLoS One. 2021 Sep 30;16(9):e0258002. doi: 10.1371/journal.pone.0258002 (PMC8483417; doi:10.1371/journal.pone.0258002)

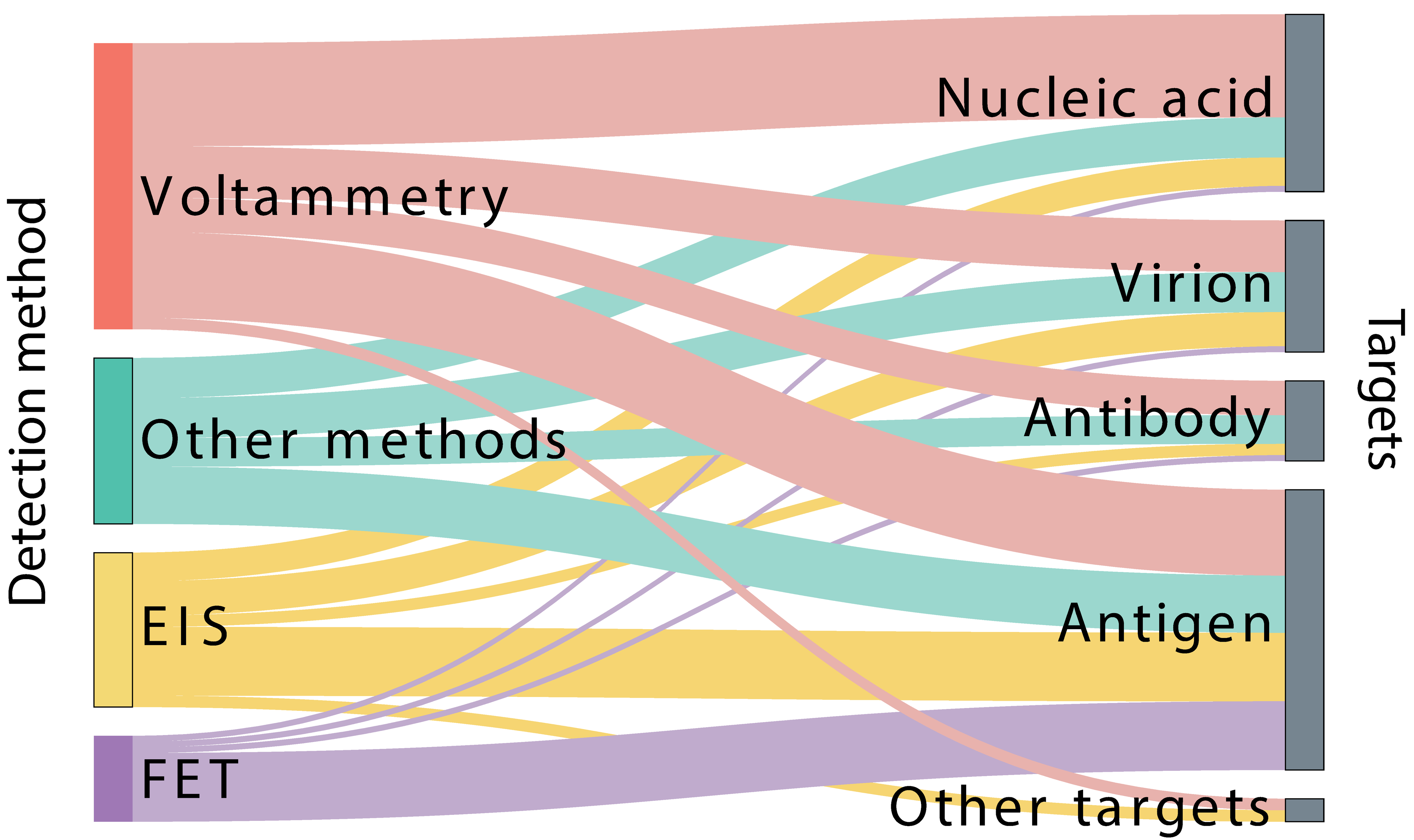

Supplement: S1 Graphical abstract — (TIFF) [file pone.0258002.s003.tiff]
